# Modulation in Wistar Rats of Blood Corticosterone Compartmentation by Sex and a Cafeteria Diet

**DOI:** 10.1371/journal.pone.0057342

**Published:** 2013-02-22

**Authors:** María del Mar Romero, Fredrik Holmgren-Holm, Maria del Mar Grasa, Montserrat Esteve, Xavier Remesar, José Antonio Fernández-López, Marià Alemany

**Affiliations:** 1 Department of Nutrition and Food Science, Faculty of Biology, University of Barcelona, Barcelona, Spain; 2 Institute of Biomedicine of the University of Barcelona, Barcelona, Spain; 3 CIBER Obesity and Nutrition, Institute of Health Carlos III, Madrid, Spain; University of Sao Paulo, Brazil

## Abstract

In the metabolic syndrome, glucocorticoid activity is increased, but circulating levels show little change. Most of blood glucocorticoids are bound to corticosteroid-binding globulin (CBG), which liver expression and circulating levels are higher in females than in males. Since blood hormones are also bound to blood cells, and the size of this compartment is considerable for androgens and estrogens, we analyzed whether sex or eating a cafeteria diet altered the compartmentation of corticosterone in rat blood. The main corticosterone compartment in rat blood is that specifically bound to plasma proteins, with smaller compartments bound to blood cells or free. Cafeteria diet increased the expression of liver CBG gene, binding plasma capacity and the proportion of blood cell-bound corticosterone. There were marked sex differences in blood corticosterone compartmentation in rats, which were unrelated to testosterone. The use of a monoclonal antibody ELISA and a polyclonal Western blot for plasma CBG compared with both specific plasma binding of corticosterone and CBG gene expression suggested the existence of different forms of CBG, with varying affinities for corticosterone in males and females, since ELISA data showed higher plasma CBG for males, but binding and Western blot analyses (plus liver gene expression) and higher physiological effectiveness for females. Good cross- reactivity to the antigen for polyclonal CBG antibody suggests that in all cases we were measuring CBG.The different immunoreactivity and binding affinity may help explain the marked sex-related differences in plasma hormone binding as sex-linked different proportions of CBG forms.

## Introduction

Glucocorticoids play a critical role in the development and maintenance of the metabolic syndrome [1]. Glucocorticoids also hamper the inflammatory immune response [2], induce insulin resistance [3], [4], enhance overall lipogenesis and fat deposition [5], and increase the liver glucose output [6], usually at the expense of amino acids [7]. Glucocorticoids waste body protein [8] and minerals [9], and there is a generalized consensus that their activity is increased in the metabolic syndrome [10]. Nevertheless, glucocorticoid excretion is more related to stress and stress-related conditions [11] than to obesity and diabetes, which usually show normal circulating serum levels [12].

Most glucocorticoids in plasma are bound/transported by a specific globulin, CBG (glucocorticoid-binding globulin) [13], a serpin with considerable homology with other members of this numerous family of proteins [14]. CBG can also bind testosterone and other hormones [15]; it is expressed in a number of tissues [16], [17], but circulating CBG is produced principally in the liver [18], [19]. There are gender differences in both humans and rats [20], [21], with females showing higher levels of the protein in serum. In obesity, CBG levels or their affinity for glucocorticoids are decreased [22]; insulin resistance and inflammation also contribute to decrease CBG levels [20], [23].

CBG, in addition to transporting glucocorticoids in plasma [24] may control or facilitate their entry in the cell [25]. CBG can bind to membrane proteins [26], and it has been found that, at least in adipose tissue, CBG may control glucocorticoid entry in the cells acting as a barrier [16]. Control of CBG has been largely attributed to regulation of its expression and release by the liver [27], but also by direct modification of the molecule and its ability to bind: i.e. the action of leukocyte elastase shortening the molecule and decreasing its effectiveness as hormone transporter [28].

In a recent study, our group has found that steroid hormones are not only transported free or bound to specific/unespecific plasma proteins, but a significant proportion of blood hormones may be carried attached to red blood cells (RBC) [29]. So far only androgen and estrogen cell/plasma distribution has been analyzed [29]. Since the case of glucocorticoids is more complex we decided to determine whether sex and/or diet, affect the compartmentation/transport of blood glucocorticoids. The objective was to find whether the known increased glucocorticoid effects observed in the metabolic syndrome [30] are a consequence of modified CBG levels [30], [31], CBG affinity [32] or compartmentation, since the changes observed in serum circulating levels or cortisol excretion could not fully explain the effects observed [10]. We used a rat model because the variables of diet, sex, age and stress could be more easily controlled, and we had access to fresh liver tissue, despite the main glucocorticoid in rodents being corticosterone and not cortisol as in humans.

## Results

### Animals

**Figure 1:**
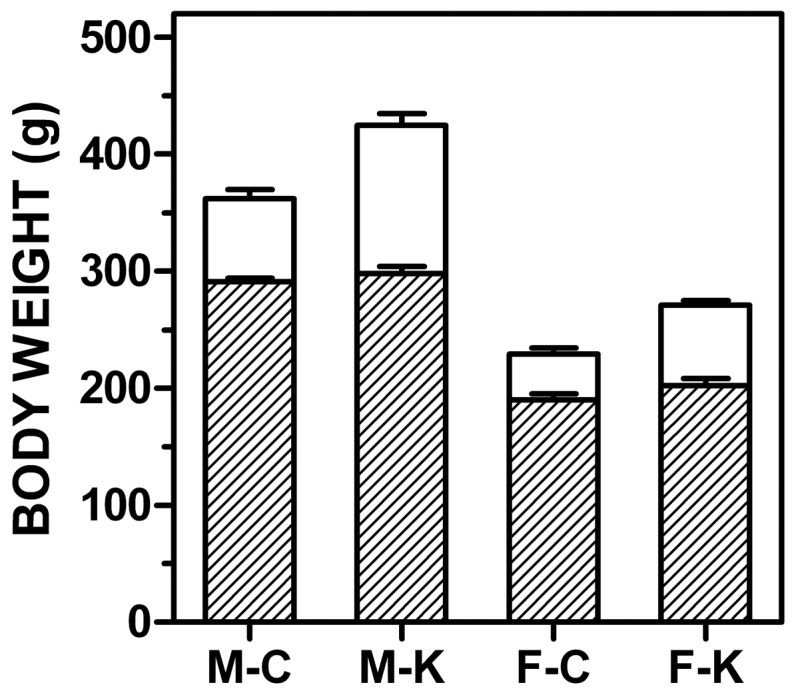
Changes in body weight of male and female rats subjected to 30 d of cafeteria diet compared with controls. The dashed columns correspond to the initial weight, and the white ones to the weight at the end of the experiment. M = male, F = female, C = control diet, K = cafeteria diet. All values are the mean ± sem of 6 different animals in each group. Statistical significance of the differences (two-way anova) showed a p<0.0001 for sex both in initial and final weights; in the final weights, there is also a significant effect of diet (p = 0.0070).

Figure 1 shows the initial and final weights of the four experimental groups. Males weighed more than females independently of diet, and increased their body weights in a higher percentage than females, the differences being more marked in the animals treated with cafeteria diet, as expected. Body weight at the end was higher than that of controls, both for males and females. Liver weight, expressed as a percentage of body weight, did not show statistically significant differences at all, but the absolute liver weights were higher in males and even higher in cafeteria-diet treated groups (data not presented). Hematocrit values were again not significantly different: 43.0±0.5 and 46.1±1.4 (males, control and cafeteria, respectively), 41.4±0.7 and 42.1±0.4 (females, control and cafeteria, respectively).

### Blood corticosterone compartmentation

Table 1 presents the levels of corticosterone in plasma (measured), free, bound to plasma proteins or to blood cells (estimated). The amount (but not the proportion) of corticosterone carried by blood cells was higher in female rats: the size of this compartment was almost twice that of free corticosterone. Bound corticosterone followed the same trend that plasma and blood corticosterone with higher values for females. In all compartments there were significant differences between groups for sex, but not for diet.

**Table 1:** Blood compartmentation of corticosterone in rats, effect of sex and diet.

parameter	units	male-control	male-cafeteria	female-control	female-cafeteria	p sex	p diet	p inter
plasma corticosterone	nM	584±108	586±91	1438±290	1099±19	0.0009	NS	NS
blood corticosterone	nM	275±52	271±39	736±156	529±72	0.0009	NS	NS
% corticosterone in blood cells	%	7.9±0.2	10.1±0.4	9.8±0.6	8.8±0.5	NS	NS	0.0017
plasma corticosterone content	nmol/L blood	253±48	244±35	661±138	482±66	0.0008	NS	NS
cells corticosterone content	nmol/L blood	22±4	27±4	75±18	47±7	0.0018	NS	NS
free corticosterone content	nmol/L blood	13±2	13±3	39±9	26±4	0.0012	NS	NS
bound plasma corticosterone	nmol/L blood	240±46	230±33	622±130	456±62	0.0008	NS	NS

The results are the mean ± sem of 6 animals per group. Statistical significance of the differences was established with a 2-way anova; inter = interaction between sex and diet.

### CBG levels

**Figure 2:**
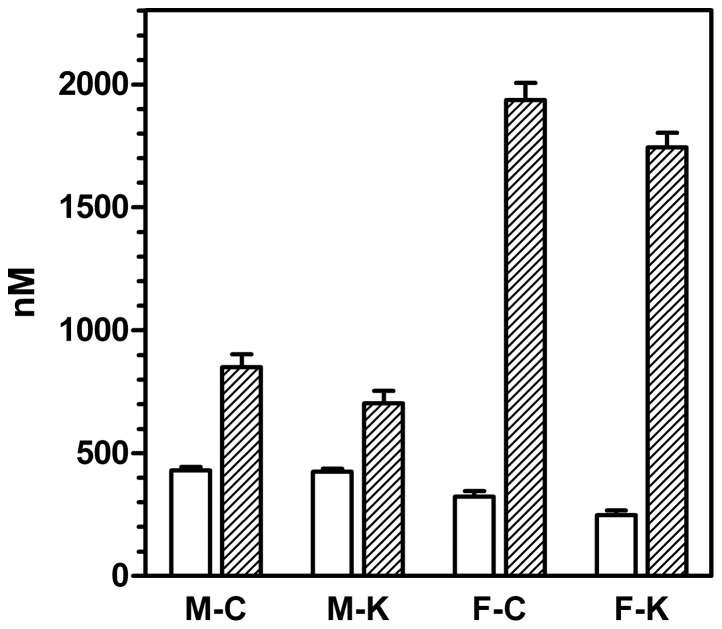
Plasma CBG levels and corticosteroid binding capacity of male and female rats subjected to 30 d of cafeteria diet compared with controls. White columns: plasma CBG levels; dashed columns: plasma corticosterone binding capacity. All values are expressed in comparable molar units, and correspond to 6 different animals per group. Abbreviations for the groups are the same as in Figure 1. Statistical significance of the differences between groups (2-way anova): There was an effect of sex (p<0.0001) and diet (p = 0.0294) for CBG levels (measured with an ELISA). Binding showed significant effects of both sex (p<0.0001) and diet (p = 0.0481).

Figure 2 shows both the levels of CBG measured by ELISA and the total corticosterone specific binding of the plasma of male and female rats. In all cases, corticosterone binding capacity was higher than the amounts of measured CBG. The levels of CBG in male rats were higher than those of females. The ELISA CBG analyses were repeated, using kits of different stocks, to analyze the same samples, and obtaining the same results. The kit was also species-specific for rat CBG (the application of the test to human or mouse samples resulted in much lower than expected levels of CBG).

**Figure 3:**
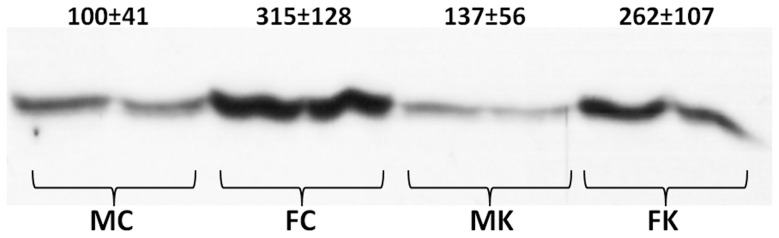
Representative Western blot of rat plasma CBG of male and female rats subjected to 30 d of cafeteria diet compared with controls. The figures written above the blot are the densitometric evaluation of 6 different animals per group, and are expressed in arbitrary units. Abbreviations for the groups are the same as in Figure 1. Statistical significance of the differences between groups (2-way anova). There was a significant (p = 0.0001) effect of sex, but not of diet (NS).

Figure 3 presents the Western blots of two animals each of the four experimental groups. The graph also shows the densitometric analysis of the spots for all the animals in each group (N = 6). Clearly, females showed a more marked (and significant) antibody response to plasma CBG than males. When comparing these data with those obtained in the ELISA measurement of plasma CBG we obtained mean quotients (nM/arbitrary units) of 4.3 and 3.1 for males (control and cafeteria, respectively), and, for females, 1.0 and 0.95. There was a consistent difference between male and female rats in the range of 3–4 fold higher values for Western *vs.* ELISA estimation of CBG.

**Figure 4:**
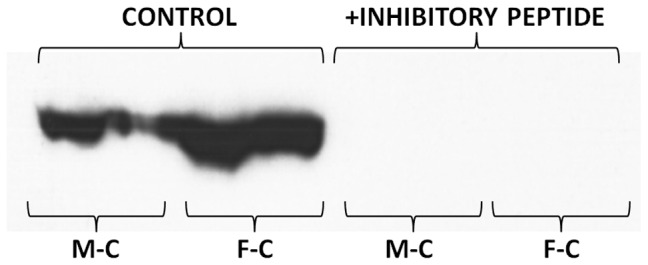
Representative Western blot of rat plasma CBG of male and female control rats in which the membranes were treated with a high concentration of the inhibitory peptide. Abbreviations for the groups are the same as in Figure 1. Addition of the peptide resulted in the loss of CBG signal both in male and female plasmas.

The presence of the inhibitory peptide blocked the binding of the primary antibody to SDS-PAGE separated proteins. No spots were detected in the membranes when the inhibitor was present (Figure 4). Similarly, the inhibitory peptide also reacted with the monoclonal antibody of the ELISA used to measure CBG. The addition of known amounts of peptide to plasma samples allowed us to establish the relationship between the binding of the monoclonal antibody to the inhibitory peptide (i.e. the antigen of the polyclonal antibody used in the Western blot) and CBG; this way, 70 nmol of the peptide were found to be equivalent to 33 nmol CBG. Large concentrations of the peptide also completely obliterated the ELISA analysis. This means that both antibodies used (ELISA's monoclonal and Western's polyclonal) recognized the representative CBG antigen, i.e. in both cases we were detecting immunoreactive protein with the structure of CBG.

**Figure 5:**
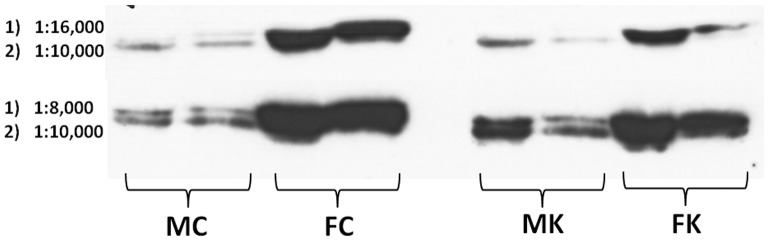
Effect of primary antibody dilution on representative Western blots of rat plasma CBG of male and female rats subjected to 30 d of cafeteria diet compared with controls. Abbreviations for the groups are the same as in Figure 1. Numbers represent the titer of the primary antibody (1) and the secondary (2). The conditions for standard Western blot used (as in Figure 4) were those of the upper photograph. In all cases, the range of MW for the spots was between 50,000 and 75,000 according to the scale (not shown, since it could be seen only in the membrane).

A revision of the conditions of development of the Western plot (i.e. increasing the titer of the primary antibody to detect possible additional CBG-like proteins) resulted in the clear manifestation of at least two immunoreactive spots, with small differences in molecular weight (Figure 5) which were discernible in males, but were completely obliterated in females. The differences in immunoreactivity of male and female plasma agree in some way with the values obtained in overall specific corticosterone binding of plasma, but not with CBG levels (ELISA). The presence of two spots could neither explain the differences, since the size of the spots in females also went in the same direction. In any case, there exists the possibility of cross-reactivity with non-CBG proteins with MW similar to that of CBG.

### Corticosterone binding; effects of testosterone

Total plasma corticosterone binding (Figure 2) was much higher in females than in males; cafeteria groups showed overall lower binding values. The ratio of CBG concentration (nM) *vs.* the maximal binding capability of plasma, also in nM (of corticosterone) was 0.51 and 0.61 in males (control and cafeteria, respectively), but only 0.17 and 0.14 in females (2-way anova, p<0.0001 for sex, NS for diet).

**Figure 6:**
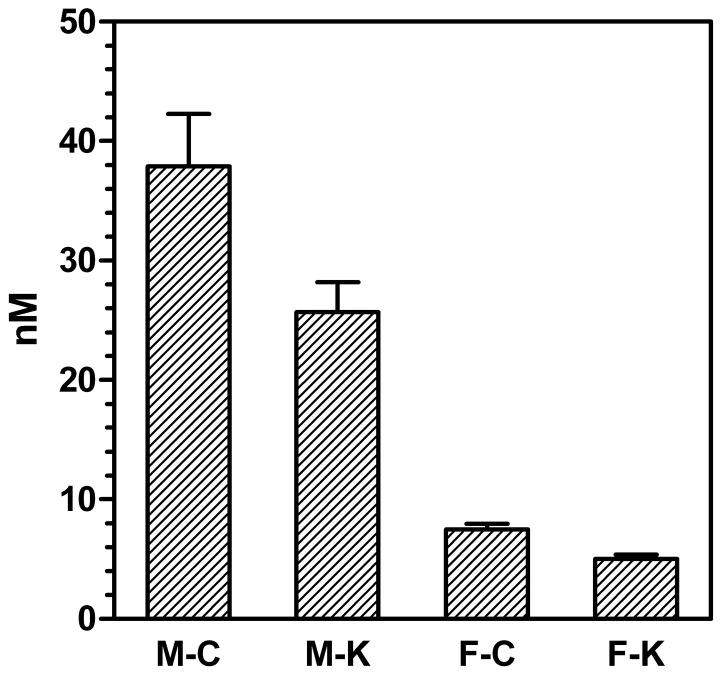
Testosterone levels in the plasma of male and female rats subjected to 30 d of cafeteria diet compared with controls. All values are expressed in comparable molar units and correspond to 6 different animals per group. Abbreviations for the groups are the same as in Figure 1. Statistical significance of the differences between groups (2-way anova); the effect of sex was significant (p<0.0001) as was that of diet (p = 0.0378).

The plasma levels of testosterone are shown in Figure 6. As expected, male had higher concentrations than female rats. Cafeteria diet decreased plasma testosterone in both sexes.

Preincubation of female plasma with testosterone did not affect significantly its ability to bind corticosterone: mean percentage of specific *vs.* total binding in the absence of testosterone (N = 6 different rats) was 95.7±0.1%, and 95.4±0.2% in its presence. The ratio of corticosterone-specific binding of testosterone-laden *vs.* control plasma was 1.16±0.07, thus testosterone pre-incubation tended to slightly decrease (but not significantly) corticosterone binding.

The binding of testosterone by plasma proteins under the conditions described in the Methods section could not be discriminated from non-specific binding. This lack of specific binding is in agreement with the lack of SHBG in rodents [33], and suggests that, even at the high concentrations tested, testosterone does not compete sufficiently with corticosterone for CBG binding sites, and thus its effects under standard conditions can be considered nil.

In female plasma, the presence of testosterone interfered, only at high (non-physiological) concentrations, the binding of corticosterone by rat plasma: the Ka for corticosterone was 16 nM (r^2^ = 0.967), and that for testosterone 2.9 µM (r^2^  = 0.880). The binding results obtained with added testosterone (irrespective of its concentration) were not different from those already shown on Table 1 for plasma corticosterone.

### CBG gene expression

Liver expression of the *Serpina6* gene (coding for CBG) was higher in females than in males, and the previous exposure to a cafeteria diet induced an additive increase on its expression; the number of transcripts per cell, thus, were about 5-fold higher in female-cafeteria than in male-control rats (Table 2). This marked difference at the cell level was maintained when considering the liver as a whole. Comparison of this capacity for CBG synthesis with the actual circulating mass of CBG resulted in two contrasting sets of data: the ratio of pmol of CBG (ELISA) in the whole plasma vs. the pmoles of mRNA*^Serpin6^* in liver were 100±22 and 60±3 for males (control and cafeteria, respectively), and 24±2 and 12±2 for females (p<0.0001 for sex and p = 0.0321 for diet, 2-way anova). However, when the binding capacity of the same plasma was compared with the liver transcript content, the results were different: 177±28 and 91±14 for males and 143±14 and 83±10 for females (NS for sex and p = 0.0006 for diet). Thus, with respect to binding capacity, there were no differences in the liver gene expression *vs.* circulating binding capacity between males and females, only diet changed (in a fairly parallel way) this ratio, decreasing the plasma binding capacity in spite of increasing the expression of this gene.

**Table 2:** CBG gene (*Serpina6*) expression in the liver of male and female rats subjected to 30 d of cafeteria diet compared with controls.

form of expression of the data	male-control	male-cafeteria	female-control	female-cafeteria	p sex	p diet	p inter
pmol mRNA*^Serpina6^*/g tissue	4.6±0.7	6.3±0.4	10.6±1.0	18.9±1.8	<0.0001	0.0001	0.0034
pmol mRNA*^Serpina6^*/whole liver	54±8	88±10	88±7	167±20	0.0002	0.0002	NS
mRNA*^Serpina6^* transcripts per cell (×10^3^)	7.1±1.1	11.8±1.0	16.6±1.0	38.6±5.2	<0.0001	0.0001	0.0051

The results are the mean ± sem of 6 animals per group. Statistical significance of the differences was established with a 2-way anova; inter = interaction between sex and diet.

## Discussion

The establishment of relationships between circulating corticosteroids (and, consequently, of CBG), pose a considerable problem because of their rhythms [34], variations in binding vs. free proportions [35], tissue interconversion between 17-hydroxy- (active) or 17-keto forms (less active) of corticosteroids, and the intrinsic problems of evaluating steroid hormones. This question has become critical in the context of the study of inflammatory processes related with the metabolic syndrome. Obesity is widely accepted to be directly related to increased glucocorticoid activity [10], but direct measurement of cortisol or corticosterone (or those of their main excretion products) do not show definite increases in their circulating levels [36]. Since we have observed the existence of an additional steroid hormone reservoir pool in blood, i.e. hormones carried on the blood cells [29], we investigated whether compartmentation played a significant role in the increased effect of glucocorticoids observed in the metabolic syndrome.

Testosterone is known to bind (with low affinity) CBG [15], but we have displaced corticosterone from its binding sites in plasma proteins only when using very high (non-physiological) concentrations. We can conclude that the differences observed in corticosterone binding effectiveness by sex could not be explained by the (limited) differences in testosterone levels or by testosterone competitive binding.

The proportion of corticosterone bound to RBCs was not specific, and was dependent on the concentration of the hormone, especially on the proportion carried by the plasma. This is in agreement with the results obtained with sex hormones [29], but in the case of corticosterone, the proportion of blood cell-carried hormone was lower, probably because corticosterone is more hydrophilic than androgens or estrogens. In spite of its relatively small size (in the range of 10% of total blood hormone), the cells' compartment was larger (twice) than that of free corticosterone.

Gender has a marked influence in blood (and plasma) corticosterone, with females showing higher values than males, as described previously [37]. These differences affect all compartments, whilst diet increased (in males) the proportion of hormone bound to blood cells; there were no other significant effects of diet on corticosterone compartmentation. These results are in line with the paradox of increased glucocorticoid effects observed in inflammation [2] with relatively unchanged plasma or serum levels [12].

The modulation of free cortisol or corticosterone is widely accepted to rest on two cooperative processes: conversion in tissues through 11β-hydroxysteroid dehydrogenases [38] and modulation of CBG levels and affinity [39], [40]. In the present study, we have observed a marked difference in the ability of CBG to bind corticosterone in females (measured CBG levels justifying about 1/6th of plasma corticosterone binding) and males (about 1/2). This difference was also observed in the hepatic expression of the gene coding for CBG (*Serpina6*), in agreement with previous studies [41]. However, the low justification of binding by CBG levels measured with a specific monoclonal antibody ELISA was also in disagreement with the analysis of CBG in plasma by Western plot using a polyclonal antibody. The differences observed between both methods were considerable. In fact, the gene expression tendency, the Western blot results and the total plasma binding capacity were in agreement: females had higher corticosterone-binding activity, CBG expression and CBG levels in plasma. But the ELISA data showed the opposite.

Thus we intended to first determine whether in both cases we were actually measuring CBG: the ELISA method was inhibited by the antigen peptide used to produce the polyclonal antibody, albeit with a lower potency (about half) than that of the complete molecule. But, evidently, the monoclonal ELISA antibody reacted fully with the antigen used to produce the antibody for Western blots. Perhaps the question was the reverse: this antibody may react to a number of other serpins [42] or bind to other non-CBG proteins. However, the inhibitory peptide suppressed all bands in the Western blots, thus showing that all were also CBG. The analysis of Western blots at a higher titer showed that probably there were (at least) two immunoreactive bands. This may be a consequence of the shortening of CBG chain by elastases [28], since the difference in molecular weight was small. However, even if that were the case, this could not explain the differences observed, since these bands were present in both females and males.

The possibility that the ELISA kit data were wrong could not be sustained, first because we repeated the test using different lots, second because the standards were purified native rat CBG, third since the ELISA antibodies were raised against a 273-peptide containing the 14-residue peptide used to raise the polyclonal antibody, and fourth because a lower response would affect both female and male, and not discriminate between them in so marked (opposite) way. The rat has a single transcript for the *Serpina6* gene and the antibody with lower overall sensitivity was that raised on the longer peptide. We checked that the 14-residue sequence used to obtain the antibody used for Western blots was unique −in rats− for CBG. Thus, both antibodies were recognizing the same protein coded sequences, i.e. CBG.

Since, in addition, not even in males the concentration of CBG justified more than 50–60% of the specific plasma binding (in itself in the range of 95% of total binding), the question remains: what protein was responsible for the remaining specifically-bound carried corticosterone? This figure is raised to perhaps 85% in the case of female rats. Our preliminary conclusion is that in plasma there may be more than one CBG isoforms, differentiated by the very specific monoclonal antibody of the ELISA kit but not by the polyclonal one: i.e. the peptide segments used to obtain them may represent a possible sequence difference between them. However, the gene was only one, thus the eventual differences should be post-translational.

Serpins are a large family of proteins [43], and a few of them are close to CBG. It is unclear whether the usual methods for gene expression and protein measurement are enough to discriminate between two possible forms of the transporter. In fact, most of the studies have been carried out using a single transcript or polyclonal antibody, generally using female rats (for their higher CBG content) or humans. However, it is highly probable that the differences between the two populations of CBG we postulate are of post-translational nature, since the data presented under Results also show that the increase in liver expression of *Serpina6* is closely related to plasma binding capacity and both are enhanced by cafeteria feeding: i.e. *Serpina6* expression is a direct response to inflammation, as are CBG [44] and glucocorticoid activity [2].

The possible existence of two forms of CBGs with different affinities for corticosteroids (and perhaps for testosterone too) may add an additional step of modulation of free corticosteroids' levels. It may be indicative that exposure to a cafeteria diet practically did not change the levels of CBG nor binding in males, but deeply altered these parameters in females. Perhaps the ultimate factors controlling this conversion are the estrogens, powerful antiinflammatory agents [45], which may act in coordination with glucocorticoids [46]. Estrogens also antagonize glucocorticoids in the development of stress or the metabolic syndrome [47], [48], and have been considered a powerful protection against the damages caused by glucocorticoids in women and female rats [49]. On the other side, males are more sensitive to glucocorticoids, which provoke hypoandrogenism [50], [51].

In conclusion, the data presented show marked sex differences in corticosterone compartmentation in rats, largely due to different affinities or forms of CBG. These differences are magnified in rats which are overweight because of exposure to a cafeteria diet and some degree of inflammation. The glucocorticoid response was intense, both changing the expression of CBG gene in liver, the levels of binding in plasma and the proportion of blood cell-bound corticosterone, but not free corticosterone. The main compartment of corticosterone in rat blood is bound to plasma proteins (essentially through specific binding), with smaller compartments of hormone bound to blood cells or free.

## Methods

### Ethics statement

All animal handling procedures were done in accordance with the norms of European, Spanish and Catalan Governments. The study was specifically approved (DMAH-5483) by the Animal Ethics Committee of the University of Barcelona

### Animals and animal handling

Wistar adult rats (9 week-old) both male and female were used (Harlan Laboratories Models, Sant Feliu de Codines, Spain). The rats were adapted to the Animal House environment for at least 7 days prior to the beginning of the experiment, and were fed the standard Harlan (type 2014) chow. Half of the rats in each group were subjected to an energy-rich limited-item cafeteria diet [52] for a month, whilst the other groups were kept as controls eating the usual rat chow. Food consumption and rat weights were recorded.

The four experimental groups (N = 6 for each) were: female-control, female-cafeteria, male-control and male-cafeteria. The animals were kept in 3-rat cages. At the end of the experiment, the rats were anaesthetized with isoflurane and killed between 1 and 2.5 hours after the beginning of the light cycle (i.e. from 9:00 to 10:30) in order to minimize the influence of circadian rhythms on corticosterone levels, and thus obtain more homogeneous and comparable data. The animals were killed by exsanguination (aortic blood drawing with a dry-heparinized syringe). Part of the blood was immediately centrifuged (at 600×g for 10 minutes at 2–4°C). Plasma was then centrifuged for 10 additional minutes at 3,000×g at 2–4°C. Plasma was frozen and kept at −20°C. Packed cells were gently resuspended in phosphate-buffered saline (PBS) pH 7.4 (12 mM phosphate buffer containing 140 mM NaCl), washed three times and used for hormone-binding assays. A second aliquot of blood was used fresh for the analysis of labeled hormone distribution, as previously described [29]. Livers were rapidly extracted, frozen with liquid nitrogen, weighed and stored at −80°C until processed.

### Hormone binding to blood cells

Fresh blood cells resuspended in PBS were used for affinity binding assays using tritium labeled corticosterone (NET39900, Perkin Elmer, Bad Neuheim, Germany, specific activity 2.12 GBq/mmol) at different concentrations (from 20 pM to 100 nM, containing 8.3 kBq/mL ^3^H-corticosterone) in 0.2 mL of the RBC suspension (i.e. about 40 mg of cells). They were incubated for 120 min at 20°C. The incubation was stopped by setting the tubes in an ice bath and immediately filtering-out the cells under vacuum through glass fiber filters (GFC Whatman, Maidstone, Kent UK). The filters were used to measure the label retained by the RBCs through scintillation counting. The addition of 200-fold cold hormone concentration to duplicates of the samples was used to differentiate specific from non-specific binding [53]. No significant specific binding of corticosterone by whole RBCs was observed in any sample.

### Plasma hormone measurement

Plasma samples were used for analysis of hormone levels in duplicate using ELISA kits. Corticosterone was measured using kit K014-H1 (Arbor Assays, Ann Arbor, MI USA), and testosterone with kit RE52151 (IBL International, Hamburg, Germany). Hormone analyses were done following the instructions of the manufacturers.

### Distribution of corticosterone in blood

The proportion of trapped plasma volume in centrifuged blood cells was considered to be 3.5% as determined previously using the same experimental setup [29].

Aliquots (0.25 mL) of fresh blood were introduced, in triplicate, in 1.5 mL Eppendorf vials; then, 5 µl of the carrier-free labeled hormone diluted in buffer (i.e. 3.7 kBq), was added to each vial using the same pipette tip for all samples. The tubes were left 2 hours in a gently shaking bath at 37°C; then were centrifuged at 16,000×g for 20 min at 4°C. Samples of supernatant plasma and packed cells were obtained, and their total weight (used to calculate their volume) and radioactivity were measured. The packed cell results were corrected for the trapped plasma volume.

The amount of hormone in RBC was estimated discounting the trapped-plasma contribution (i.e. 3.5% of packed cell volume corresponding to plasma, which hormone concentration we had measured). The ratio of RBC versus plasma label distribution (r) was measured as previously described [29]. This allowed us to obtain the RBC corticosterone content:
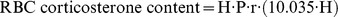
where “P” was the plasma hormone concentration, 0.035 the fraction of plasma trapped in packed cells, and “H” was the hematocrit fraction (Hc/100). In consequence, the contribution of plasma to total blood hormone is the sum of the hormone found in plasma plus that trapped between packed cells [29]. Since all these parameters were available for each individual rat, we could estimate the blood hormone concentration for each rat of the four groups. The proportion of hormone in each of the three compartments (free, plasma-bound and cell-bound) was also calculated for each individual rat.

The free corticosterone fraction was determined by ultrafiltration of plasma [29]. The samples were centrifuged, using Centrifree tubes (Millipore Ireland, Carrigywohill, Ireland), at 1500×g for 20 minutes at 30°C, and the ultrafiltrate radioactivity was measured.

### Plasma corticosterone binding

Samples of plasma (10 µL) were mixed with 0.500 mL of a suspension of dextran-charcoal (0.5 g/L and 5 g/L, respectively) in PBS pH 7.4 containing 1 g/L gelatine [54]. The tubes were left at room temperature for 30 min with occasional shaking and then centrifuged 15 min at 4°C and 4,500×g. This procedure ensured the elimination of the steroid hormones in plasma [54].

Supernatants were diluted 1∶4 with chilled PBS-gelatine. Total binding was estimated using 0.150 mL of diluted supernatant, 0.025 mL of PBS-gelatine, and 0.025 mL of ^3^H-labelled corticosterone (final concentration 15 nM, specific radioactivity 12 kBq/nmol). Non-specific binding was estimated under the same conditions, but now adding 0.025 mL of non-labelled corticosterone (final concentration 7.5 µM). In both cases, the tubes were incubated under gentle shaking for 20 min at 37°C followed by 2 h at 4°C. Then 0.200 mL of chilled dextran-charcoal PBS-gelatine buffer were added, and the tubes were vortexed and kept in the cold 10 min. Finally, the tubes were centrifuged 10 min at 4°C and 2,000×g. Aliquots of the supernatants were used to measure the radioactivity bound to proteins.

### Measurement of CBG levels in plasma

Plasma CBG levels were measured using a specific (monoclonal antibody raised on the 23–296 amino acids fragment of CBG) rat CBG ELISA kit (E91226Ra, USCN, Wuhan, China) against a rat CBG standard.

A rough estimation of the mass of circulating CBG was calculated from the data of CBG plasma concentration (ELISA), the hematocrit value and the approximate proportion of blood *vs.* body weight in mammals (5%). The same approach was used to estimate the overall plasma capacity for corticosteroid binding.

### CBG Western blot

Plasma CBG was also estimated by Western blot using a polyclonal antibody: EB10756 goat anti transcortin/CBG (rat) (Everest Biotech Upper Heyford, Oxfordshire UK) raised against a representative peptide (EBP10756 immunizing peptide, Everest Biotech). The presence of this peptide in the system blocked practically all the primary antibody, thus we used it as an inhibitory peptide. The peptide sequence was C-KASQQINQHVKDKT. The secondary antibody was rabbit antigoat Ig-HRPsc-2922 mouse/human adsorbed (Santa Cruz Biotechnology, Santa Cruz, CA USA). Proteins were run by SDS-PAGE (7.5% resolving gel; 4% stacking gel). A protein MW marker scale (kaleidoscope, BioRad, Berkeley, CA USA) was included in one of the wells. Samples of 25 µg protein (Bradford [55]) of plasma were used. Proteins were transferred to PVDF Immobilon (Millipore, Billerica, MA USA) membranes, which were visualized with Ponceau red. The membranes were blocked with 5% TBST and skimmed milk; then the membranes were incubated with the primary antibody (1∶16,000 at 4°C overnight), washed four times and then exposed to the secondary antibody 1∶15,000 for 1 h at room temperature. After four washings, chemiluminiscence was analyzed using Luminata-Crescendo (Millipore) for 2 min. Then densitometric measurements were done using the Total-Lab program (Nonlinear USA, Durham, NC USA). This Western blot has been previously used by our group for the measurement of plasma CBG [56].

The presence of large concentrations of the inhibitory peptide competed with native plasma CBG, which resulted in the disappearance of any binding in Western membranes.

### Testosterone interference with CBG binding

Since testosterone binds CBG [15], we investigated whether testosterone interfered the binding of corticosterone by rat blood plasma by carrying out three experiments using the plasma of the six control female rats. It was assumed that the higher levels of testosterone in male rats may have already affected their plasma corticosterone binding, and thus female rats (with lower testosterone) would be more sensitive to an overload of the androgen.

In the first test, a binding analysis was carried out as described above to measure the binding of testosterone to female rat plasma, in the presence of testosterone at concentrations ranging from 0.1 pM to 1 µM.

In the second test, plasma samples were incubated in the presence or absence of added 100 nM testosterone at 30°C for 2 h. It was expected that previous “saturation” of plasma with free unlabeled testosterone may later decrease the binding ability of CBG, and then corticosterone binding was carried out as described above.

The third test consisted in carrying out a standard corticosterone binding assay in the presence or absence of just added 100 nM testosterone, i.e. without prior incubation with the androgen.

### Estimation of liver CBG gene expression

Total tissue RNA was extracted from the frozen liver samples using the Tripure reagent (Roche Applied Science, Indianapolis IN USA), and were quantified in a ND-100 spectrophotometer (Nanodrop Technologies, Wilmington DE USA). RNA samples were reverse transcribed using the MMLV reverse transcriptase (Promega, Madison, WI USA) system and oligo-dT primers.

Real-time PCR (RT-PCR) amplification was carried out using 10 µL amplification mixtures containing Power SYBR Green PCR Master Mix (Applied Biosystems, Foster City, CA USA), 8 ng of reverse-transcribed RNA and 300 nM of primers. Reactions were run on an ABI PRISM 7900 HT detection system (Applied Biosystems) using a fluorescent threshold manually set to OD 0.5 for all runs. Cycling times were used for the semiquantitative determination of the number of the CBG gene (*Serpina6*) transcripts in the sample as previously published [57]. In order to determine the sensitivity and efficiency of the amplification process, PCR linearity ranges were previously established for the gene with tissue cDNA. The primers used for the *Serpina6* expression measurement were: 3′-5′ CCTCCTTCATCCTGGTCAAC, and 5′-3′ TTCCATTTCCCACATAGTCCA. As housekeeping gene we used cyclophilin (*Cypa*): 3′-5′ CTGAGCACTGGGGAGAAAGGA, 5′-3′ GAAGTCACCACCCTGGCACA.

Liver DNA content was measured using a colorimetric method [58]. The content of mRNA*^Serpina6^* was related to unit of liver weight, total liver weight (i.e. an estimation of overall liver capacity for CBG production) and as number of transcripts per cell [57], by assuming a mean DNA cell content of 5.60 pg [59] and the measured amount of DNA per g of liver.

### Statistical analysis

Statistical analysis was carried out using two-way anova analyses (sex, diet), using the Statgraphics Centurion XVI program package (Statpoint Technologies, Warrengton VA USA).
